# Trends in HIV pre-exposure prophylaxis uptake in Ontario, Canada, and impact of policy changes: a population-based analysis of projected pharmacy data (2015–2018)

**DOI:** 10.17269/s41997-020-00332-3

**Published:** 2020-06-11

**Authors:** Darrell H. S. Tan, Thomas M. Dashwood, James Wilton, Abigail Kroch, Tara Gomes, Diana Martins

**Affiliations:** 1 Division of Infectious Diseases, St. Michael’s Hospital, Toronto, ON Canada; 2 MAP Centre for Urban Health Solutions, St. Michael’s Hospital, 30 Bond St., Toronto, ON M5B 1W8 Canada; 3 Department of Medicine, University of Toronto, Toronto, ON Canada; 4 Li Ka Shing Knowledge Institute, St. Michael’s Hospital, Toronto, ON Canada; 5 Ontario HIV Treatment Network, Toronto, ON Canada; 6 Leslie Dan Faculty of Pharmacy, University of Toronto, Toronto, ON Canada

**Keywords:** Pre-exposure prophylaxis, HIV, Policy, Public health, Prevention and control, Prophylaxie pré-exposition, VIH, politique (principe), santé publique, prévention et contrôle

## Abstract

**Objectives:**

HIV pre-exposure prophylaxis (PrEP) is a proven tool for HIV prevention, but PrEP use in Ontario, Canada, and the effects of recent policies are unknown. We estimated the number and characteristics of PrEP users in Ontario and evaluated the impacts of policy changes between July 2015 and June 2018.

**Methods:**

We obtained tenofovir disoproxil fumarate/emtricitabine (TDF/FTC) dispensation data for Ontario from IQVIA, and applied an algorithm to identify use for PrEP. We report prevalent PrEP use for the second quarter of 2018 according to age, sex, region, prescriber specialty, and payer type, and generate “PrEP-to-need ratios” (PNR) by dividing these numbers by the estimated numbers of new HIV diagnoses. We used interventional autoregressive integrated moving average models to examine the impact of three policy changes on PrEP use: Health Canada approval (February 2016), availability of generic TDF/FTC and partial public drug coverage (September 2017), and public drug coverage for individuals aged < 25 years (January 2018).

**Results:**

The estimated number of individuals receiving PrEP increased 713%, from 374 in 2015 Q3 to 3041 in 2018 Q2. Among PrEP users in 2018 Q2, 97.5% were male, 60.4% were < 40 years, 67.7% obtained PrEP from a family physician, 77.2% used private insurance, and 67.0% were in Toronto. PNRs were highest in 30–39-year-olds, males, Toronto and the Central East and West regions. Time series analyses found that Health Canada approval (*p* = 0.0001) and introducing generics/partial public drug coverage (*p* = 0.002) led to significantly increased use.

**Conclusions:**

PrEP use has risen in Ontario in association with favourable policy changes, but remains far below guideline recommendations.

**Electronic supplementary material:**

The online version of this article (10.17269/s41997-020-00332-3) contains supplementary material, which is available to authorized users.

## Résumé

**Objectifs:**

La prophylaxie pré-exposition (PPrE) est un outil éprouvé pour prévenir le VIH, mais le recours à la PPrE en Ontario (Canada) et les effets de politiques récentes sont inconnus. Nous avons estimé le nombre et les caractéristiques des utilisateurs de la PPrE en Ontario et évalué les incidences de changements de politique survenus entre juillet 2015 et juin 2018.

**Méthode:**

Nous avons obtenu auprès d’IQVIA des données sur l’administration de fumarate de ténofovir disoproxil (FTD) et d’emtricitabine (FTC) en Ontario et appliqué un algorithme pour déterminer le recours à la PPrE. Nous présentons la prévalence du recours à la PPrE au deuxième trimestre de 2018 selon l’âge, le sexe, la région, la spécialité du médecin prescripteur et le type de payeur, et nous générons des « ratios PPrE-besoins » (RPB) en divisant ces nombres par les nombres estimatifs de nouveaux diagnostics de VIH. Nous avons utilisé des modèles interventionnels fondés sur la moyenne mobile intégrée autorégressive pour examiner les incidences de trois changements de politique sur le recours à la PPrE : l’approbation par Santé Canada (février 2016); la disponibilité de versions génériques du FTD et de la FTC et leur couverture partielle par le régime public d’assurance-médicaments (septembre 2017); et la couverture des moins de 25 ans par le régime public d’assurance-médicaments (janvier 2018).

**Résultats:**

Le nombre estimatif de personnes recevant la PPrE a augmenté de 713 %, passant de 374 au troisième trimestre de 2015 à 3041 au deuxième trimestre de 2018. Chez les utilisateurs de la PPrE au deuxième trimestre de 2018, 97,5 % étaient des hommes, 60,4 % avaient moins de 40 ans, 67,7 % obtenaient la PPrE auprès d’un médecin de famille, 77,2 % utilisaient une assurance privée, et 67,0 % vivaient à Toronto. Les RPB les plus élevés ont été observés chez les 30 à 39 ans, chez les hommes et chez les résidents de Toronto et des régions du Centre-Est et du Centre-Ouest. Selon les résultats d’analyses des séries chronologiques, l’approbation par Santé Canada (*p* = 0,0001) et l’introduction de versions génériques/la couverture partielle par le régime public d’assurance-médicaments (*p* = 0,002) ont entraîné des hausses significatives du recours à la PPrE.

**Conclusions:**

Le recours à la PPrE a augmenté en Ontario en lien avec des changements de politique favorables, mais il demeure très en-deçà des recommandations des lignes directrices.

## Introduction

HIV prevention remains a priority in Ontario, with new diagnoses remaining stable for the past several years (Ontario HIV Epidemiology and Surveillance Initiative 2019). Pre-exposure prophylaxis (PrEP), with regular oral tenofovir disoproxil fumarate and emtricitabine (TDF/FTC), is a safe, efficacious intervention that decreases HIV risk by over 99% if adherence is high, making it a potential “game-changer” in the fight against HIV (Grant et al. 2010; Choopanya et al. 2013). The first trial that demonstrated PrEP efficacy was conducted in gay, bisexual, and other men who have sex with men (gbMSM) in 2010 and demonstrated a 92% reduction in HIV incidence in participants with detectable TDF/FTC levels compared with controls (Grant et al. 2010). In heterosexual adults, several trials have demonstrated PrEP efficacy: TDF2 showed a 62% reduction in HIV incidence in adults taking TDF/FTC compared to placebo (Thigpen et al. 2012). The Partners PrEP study showed a relative risk reduction of 90% in those with detectable levels of TDF or TDF/FTC compared with placebo (Baeten et al. 2012). In adults who inject drugs, daily tenofovir use with detectable drug levels showed a risk reduction of 74% (Choopanya et al. 2013). The IPERGAY trial demonstrated that TDF/FTC can also be taken “on-demand” as PrEP, and reduce HIV risk by 85% among MSM (Molina et al. 2015). Of note, adherence with study drug in these trials is known to have been suboptimal.

TDF/FTC became available for HIV treatment in Canada in 2006, and off-label use as PrEP likely began shortly after the first positive trials were published (Health Canada 2018). However, use of PrEP has historically been limited due to lack of regulatory approval, suboptimal awareness, modest numbers of providers, cost, and other factors. At least four recent changes have made PrEP more accessible in Ontario. First, Health Canada granted regulatory approval for PrEP in February 2016, a pre-requisite for public or private reimbursement. Second, in August/September 2017, three generic versions of TDF/FTC became available in Canada, leading to a fourfold decrease in medication costs. Third, in September 2017, Ontario added PrEP to the list of drugs covered through the Ontario Drug Benefit (ODB) program. This program provides free drug coverage for individuals on social assistance, those residing in long-term care homes, recipients of home care, and those aged ≥ 65; other Ontario residents can also access the ODB program with the payment of an annual deductible scaled to household income (Government of Ontario 2020). We classify PrEP as “partially” publicly funded because of the latter requirement for payment of a deductible. In addition, on January 1, 2018, Ontario expanded public drug coverage through the ODB program to include all Ontarians < 25 years old, through a program called OHIP+ (Government of Ontario 2020).

A major gap in our ability to monitor the success of PrEP implementation efforts in Ontario is the lack of data on population-level PrEP use, which is essential for evaluating targeted policy changes and estimating the impact on the HIV epidemic. To address this need, we leveraged pharmacy dispensation data to achieve three main objectives. The first objective was to estimate population-level PrEP use in Ontario between 2015 and 2018. The second was to compare estimates of PrEP use, defined as prevalent use for a specified time period, to HIV diagnoses according to key demographic variables. The third objective was to evaluate the impact of the four major policy changes, detailed above, on the number of PrEP users in Ontario.

## Methods

### Data source

We obtained pharmacy prescription dispensing data from IQVIA, a private pharmaceutical informatics company. IQVIA used its retail pharmacy drug dispensation database, which draws on > 2000 pharmacies representing approximately 64% of all dispensed retail prescriptions in Ontario, excluding hospital-operated pharmacies not open to the general public, to quantify TDF/FTC use in the province from July 1, 2015, to June 30, 2018. Only 3 years of data were available due to financial constraints, and we chose this period to ensure an adequate data for time series analyses. IQVIA then extrapolated the sample to the provincial level using proprietary weighting methods. Weights are derived from several measures including sales data from pharmaceutical companies regarding the total number of prescriptions in the province, the number of pharmacies in a region, the distance between IQVIA-captured and uncaptured pharmacies, and the size of the pharmacies. To provide a measure of precision for these estimates, IQVIA undertook a validation against its census sales database for Ontario, which measures the actual sales of pharmaceutical products sold indirectly through wholesalers and chain warehouses, as well as directly from the manufacturers, to retail pharmacies. Estimates were validated within 2% for the total antiretroviral market overall (within 1% for brand name products and within 8% for generic products). Individuals were linked by IQVIA using identifiers available at the pharmacy level in order to know that separate dispensation events were related to the same person.

**Fig. 1:**
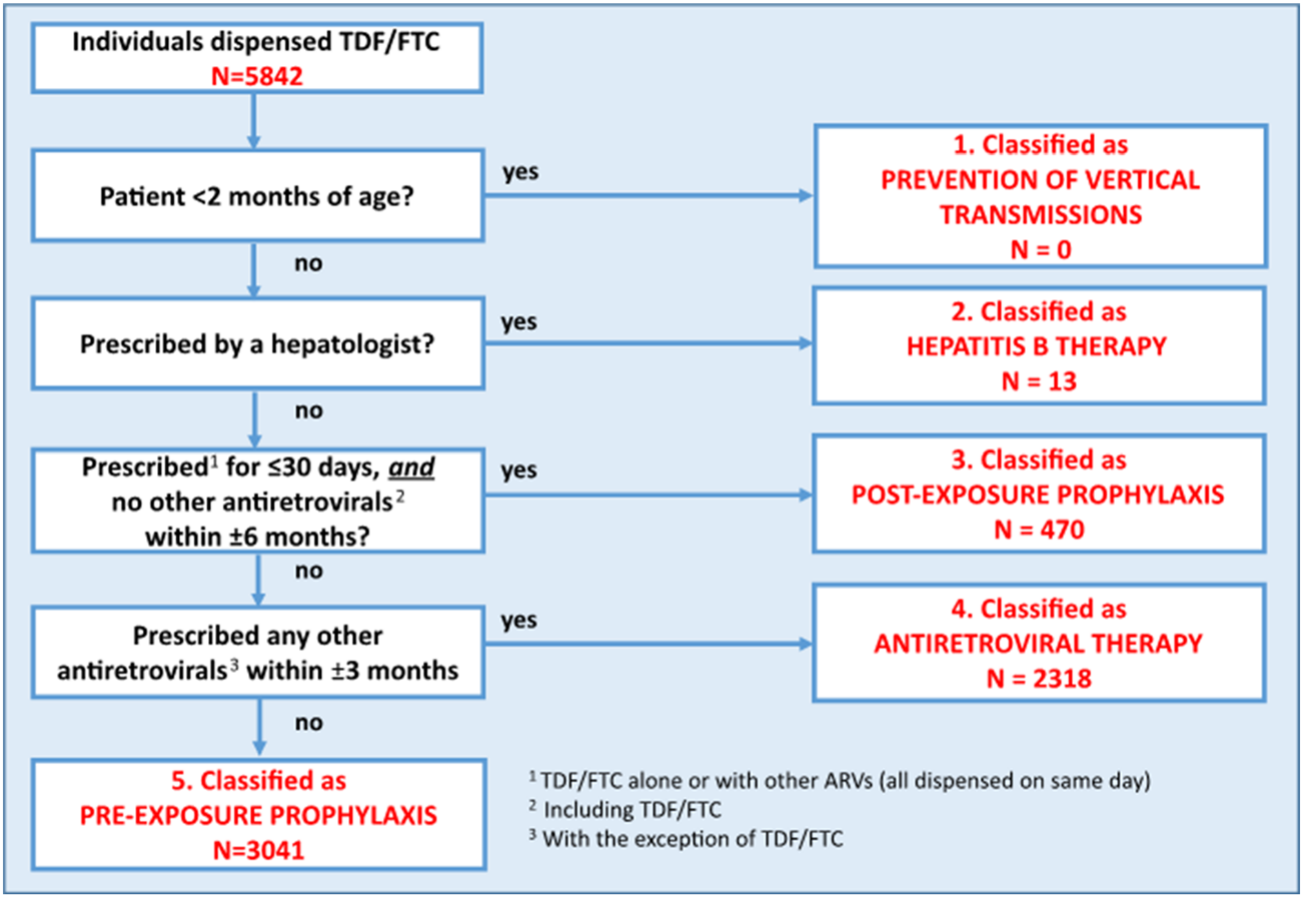
TDF/FTC dispensations and inferred clinical indications, April 2018–June 2018. Estimated number of individuals dispensed TDF/FTC in Ontario during 2018 Q2, classified by clinical indication. TDF/FTC = tenofovir disoproxil fumarate and emtricitabine

Because the clinical indications for prescriptions are not captured in IQVIA data, we developed an algorithm to infer which TDF/FTC prescriptions were for PrEP (Fig. 1). We assumed that (1) TDF/FTC prescriptions by a gastroenterologist represented treatment for hepatitis B; (2) TDF/FTC prescriptions for ≤ 30 days with no other antiretrovirals dispensed within ± 6 months represented post-exposure prophylaxis; and (3) TDF/FTC prescriptions with additional antiretrovirals dispensed within ± 3 months represented HIV treatment. The time window criteria for antiretroviral prescriptions were selected based on clinical reasoning, and the algorithm is similar to that developed by other authors (Wu et al. 2017; Mera-Giler et al. 2015).

IQVIA applied this algorithm to generate monthly and quarterly estimates of the total number of unique individuals dispensed PrEP at least once over that time period, and stratified estimates according to age, sex, geographic location (based on the postal code of the dispensing pharmacy and dividing Ontario into 14 regions based on aggregations of public health units; Table S2), payer type (public or private), and prescriber specialty.

### Statistical analysis

To achieve our first objective, we used descriptive statistics to estimate the number of individuals using PrEP in Ontario. For this objective, we used quarterly time periods, since most individuals on PrEP are dispensed TDF/FTC every 3 months. For the most recent quarter in the study period (April 2018–June 2018), we present the estimated number of PrEP users in the province and their distribution according to age, sex, geographic location, drug coverage type, and prescriber specialty.

For our second objective, we compared the estimates of PrEP users to the number of new HIV diagnoses in Ontario, where possible, using surveillance data (Ontario HIV Epidemiology and Surveillance Initiative 2017; Ontario HIV Epidemiology and Surveillance 2018). Consistent with existing literature (Siegler et al. 2018; Sullivan et al. 2018), we calculated a “PrEP-to-need ratio” (PNR) by dividing the estimated number of PrEP users in 2018 Q2 by the number of new HIV diagnoses in 2017 (since this was the period for which the most recent HIV diagnosis data were available) for a specific stratum.

To achieve our third objective, we fit an interventional autoregressive integrated moving average (ARIMA) model to the monthly number of individuals receiving PrEP in Ontario. Intervention time series analyses are commonly used to test the impact of introducing new policies on time series trends, with the null hypothesis of no effect of the intervention on trends (Gomes et al. 2017; Guan et al. 2018). We differenced the time series to achieve stationarity and confirmed this using the augmented Dickey-Fuller test. We selected model parameters based on the residual autocorrelation function (ACF), partial autocorrelation function (PACF), and inverse autocorrelation function (IACF) correlograms. Final model selection was confirmed using the autocorrelation plots, the Ljung-Box chi-square test for white noise, and *r*-square estimate of fit. We added intervention functions to the model at each intervention time point of interest. A ramp intervention function was used to test for gradual changes in trends and a step intervention function was used to test for immediate changes, based on visual inspection of the time series. The interventions included were February 2016 (Health Canada approval), September 2017 (introduction of generics and ODB coverage), and January 2018 (OHIP+ coverage). While the publication of Canadian Guidelines on PrEP in November 2017 was originally considered for inclusion in our analyses, this event was omitted from the final models because it occurred close in time to the other events; hence, its effects could not be disentangled from those policies introduced in September 2017. The *t*-statistic from the maximum likelihood estimation was used to determine if the intervention functions were significant parameters in the ARIMA model and thus had a significant impact on the time series. Final model specifications can be found in Table S1. We used a type 1 error rate of 0.05 and conducted the analyses using SAS statistical software (v 9.3, EG 6.1; SAS Institute, Cary, NC) and the SAS/ETS Time Series Forecasting System.

Ethics approval and informed written consent were not required because the data retrieved from IQVIA were de-identified, aggregated estimates.

## Results

**Fig. 2:**
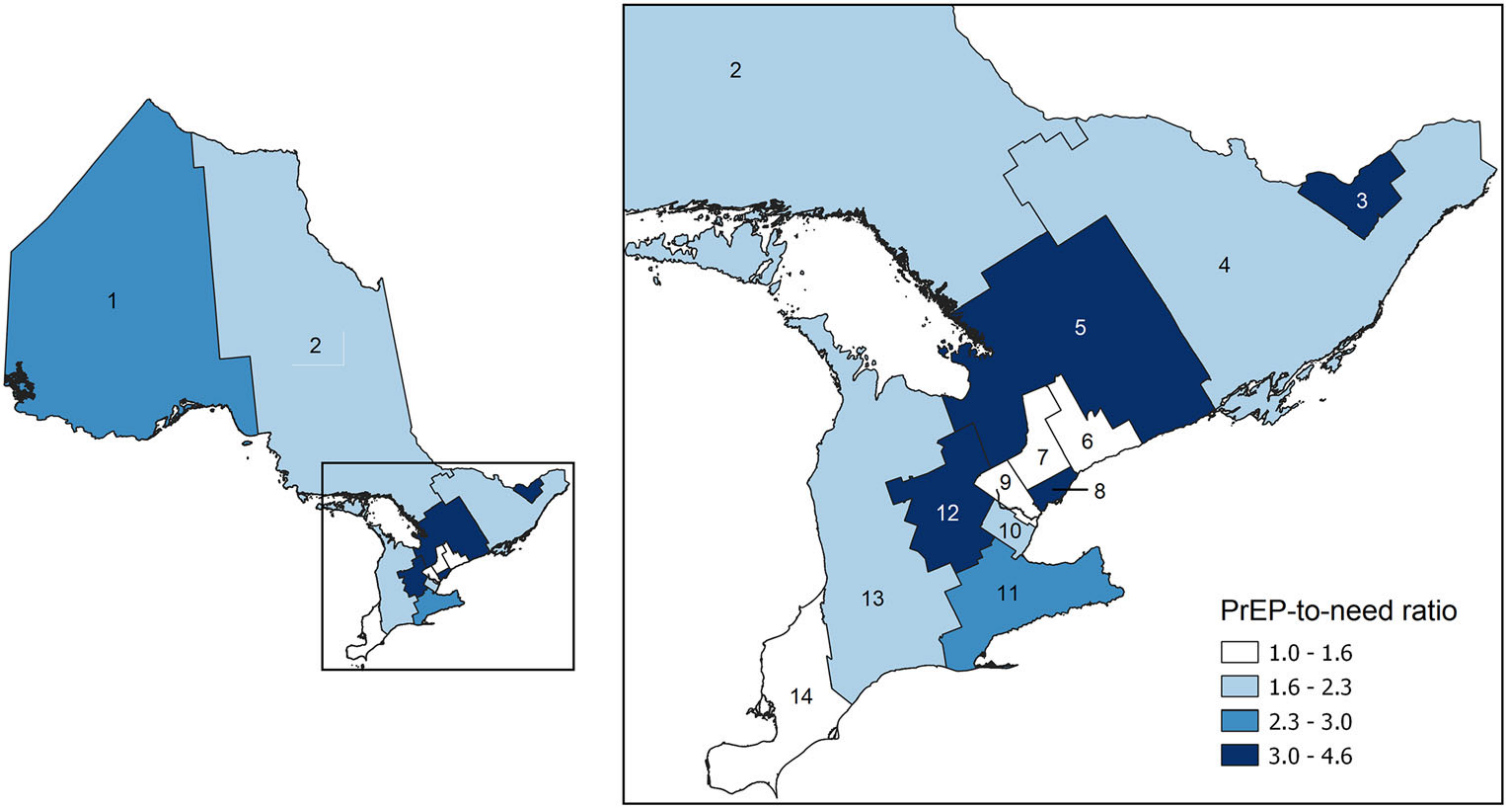
PrEP-to-need ratio by geographic region in Ontario. Ratio calculated using 2018 Q2 PrEP data and 2017 new HIV diagnosis data. 1 = North West, 2 = North East, 3 = Ottawa, 4 = Eastern, 5 = Central East, 6 = Durham, 7 = York, 8 = Toronto, 9 = Peel, 10 = Halton, 11 = Central South, 12 = Central West, 13 = South West, 14 = Erie-St.Clair. Regions based on (aggregations of) Public Health Unit (PHU) boundaries (see Table S2). Maps created using Statistics Canada boundary files (Statistics Canada 2015)

Over the 3-year study period, the estimated number of individuals using PrEP increased by 713%, from 374 in 2015 Q3 to 3041 in 2018 Q2. The number of individuals on PrEP in 2018 Q2 was equivalent to a prevalence of approximately 21.4 users per 100,000 people in Ontario. Among the PrEP users in 2018 Q2, a majority were male (97.5%), < 40 years old (60.4%), prescribed PrEP by a family physician or general practitioner (67.7%), covered through private health insurance (77.2%), and dispensed PrEP from a pharmacy in Toronto (67.0%). Further descriptive analyses are summarized in Table 1 and Fig. 2. Trends by payer type and prescriber specialty are shown in Supplemental Fig. S1. Of note, the proportion of prescribers who were infectious disease physicians decreased over the study period (from 21.9% to 13.8%) and the proportion of users who accessed publicly funded PrEP increased from 2017 Q3 onwards (from 13.3% to 22.8%).

**Table 1:** Comparison of PrEP use to HIV diagnoses by sex, age, and geographic region, Ontario

	PrEP		HIV diagnoses		PNR
	*n*	%	*n*	%	
Sex					
Male	2951	97.5	717	78.6	4.1
Female	77	2.5	195	21.4	0.4
Unknown	10		4		
Age					
< 19	17	0.6	21	2.3	0.8
19–24	281	9.2	91	9.9	3.1
25–29	457	15.0	151	16.5	3.0
30–39	1080	35.5	278	30.4	3.9
40–45	435	14.3	127	13.9	3.4
46–49	245	8.1	68	7.4	3.6
50–59	386	12.7	123	13.4	3.1
60–64	64	2.1	27	3.0	2.4
65+	73	2.4	29	3.2	2.5
Unknown	0		1		
Region					
Central East	46	1.5	10	1.1	4.6
Central South	158	5.2	54	6.0	2.9
Central West	79	2.6	19	2.1	4.2
Durham	19	0.6	15	1.7	1.3
Eastern	42	1.4	19	2.1	2.2
Erie-St.Clair	57	1.9	35	3.9	1.6
Halton	32	1.1	14	1.5	2.3
North East	22	0.7	12	1.3	1.8
North West	27	0.9	9	1.0	3.0
Ottawa	290	9.6	77	8.5	3.8
Peel	64	2.1	64	7.1	1.0
South West	114	3.8	51	5.6	2.2
Toronto	2016	67.0	496	54.7	4.1
York	44	1.5	31	3.4	1.4

PrEP numbers based on 2018 Q2 data and HIV diagnosis numbers based on 2017 data. Geography assigned based on location of dispensing pharmacy. *PNR*, PrEP-to-need ratio; *Q*, calendar quarter; *PrEP*, pre-exposure prophylaxis

A comparison of PrEP use to new HIV diagnoses along with PNRs is shown in Table 1 and Fig. 2. The numbers of PrEP users and HIV diagnoses were both highest in males (2951 users; 717 diagnoses), the city of Toronto (2016 users; 496 diagnoses), and the 30–39 age category (1080 users; 278 diagnoses). The overall PNR was 3.4 and, when stratified, was highest in the Central East region (4.6), Central West region (4.2), Toronto (4.1), males (4.1), and the 30–39 age category (3.9). PNRs were lowest in females (0.1), < 19 age category (0.8), and in the regions of Peel (1.0), York (1.4), and Durham (1.3), all of which are neighbouring regions to the city of Toronto.

**Fig. 3:**
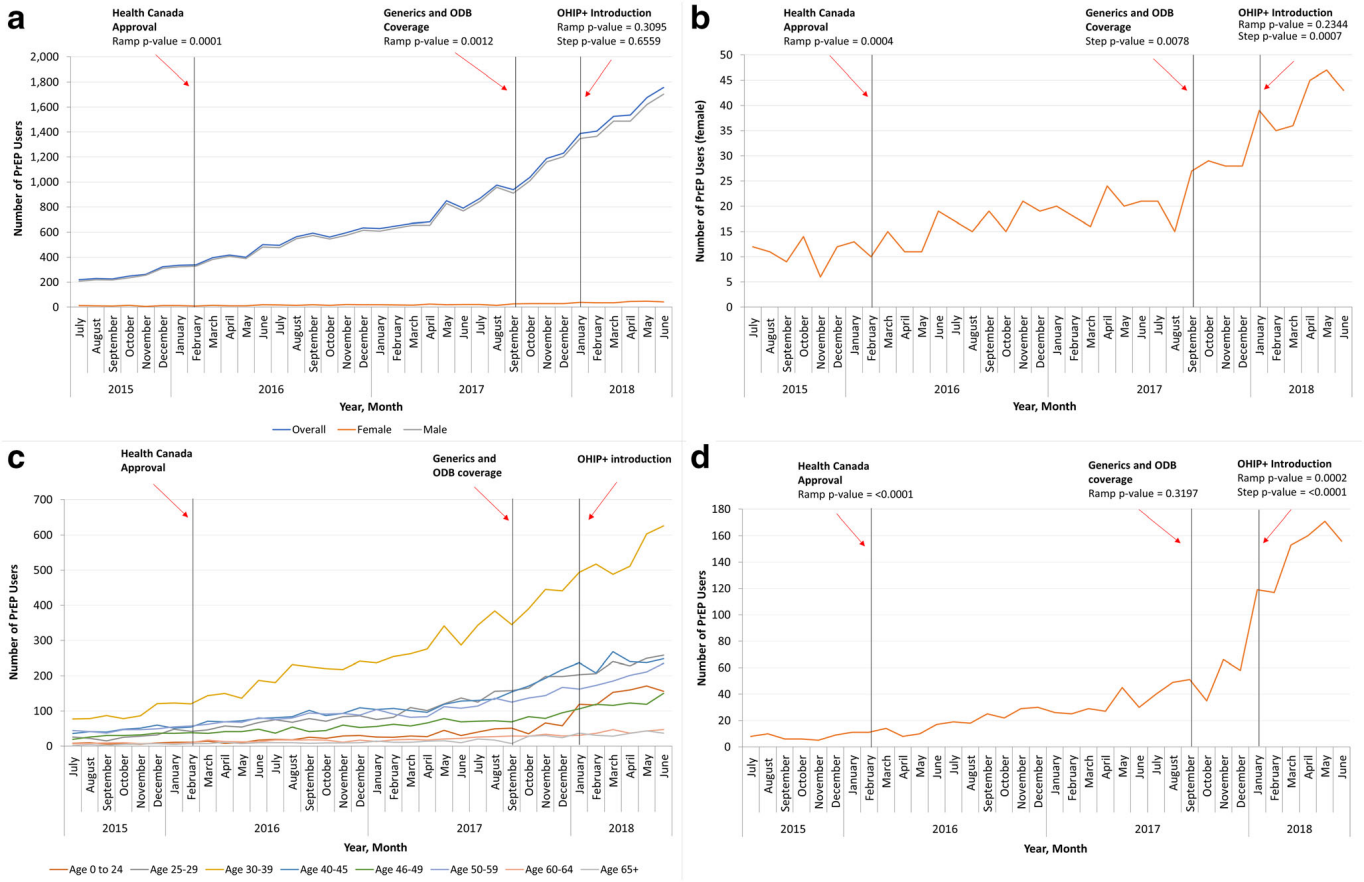
Estimated number of PrEP users over time in Ontario by sex (**a**), among females (**b**), by age (**c**), and among individuals aged < 25 years (**d**) and intervention time series analysis. Estimates were obtained using an autoregressive integrated moving average model, with intervention time series analysis of Health Canada approval of PrEP (February 2016), introduction of generic and ODB coverage (September 2017), and introduction of OHIP+ (January 2018). A ramp intervention function was used to test for gradual changes in trends and a step intervention function was used to test for immediate changes, based on visual inspection of the time series. Estimates were based on the monthly number of individuals using PrEP in Ontario between 2015 Q3 and 2018 Q2. PrEP = pre-exposure prophylaxis. ODB = Ontario Drug Benefit. OHIP+ = Ontario Health Insurance Plan Plus

The estimated monthly number of individuals filling a prescription for PrEP in Ontario increased 692% over the study period, from 219 in July 2015 to 1735 in June 2018. Overall, we found statistically significant increases in this number following regulatory approval of TDF/FTC for PrEP indications by Health Canada in February 2016 (*p* = 0.0001), and following the availability of generic PrEP medication and introduction of PrEP onto Ontario’s public drug program in August/September 2017 (*p* = 0.0012; Fig. 3a). In addition, the expansion of public drug coverage for Ontarians aged ≤ 24 in January 2018 was associated with significant increases in the number of PrEP users both among individuals in this age category, as well as in females overall (Fig. 3b–d). Model results are presented in a Supplemental Table, S2.

## Discussion

To our knowledge, this is the only study to quantify the number of individuals using PrEP in Ontario and the first to investigate how key policy changes introduced at the provincial and national level have impacted PrEP use. Over our 3-year study period, we found a 713% increase in PrEP users, with approximately 3000 individuals using PrEP in the province by mid-2018. However, this remains far below the total number of individuals in Ontario who likely meet the Canadian guideline recommendations for PrEP use, which is preliminarily estimated at > 30,000 for gbMSM alone (Tan et al. 2017a, b). Local estimates for other priority populations have not been generated. We also found that key policy changes designed to improve medication access led to increases in PrEP use over time, and that use was generally concentrated in populations experiencing the highest rates of HIV diagnoses. Our findings have important implications for jurisdictions where PrEP rollout has been slow and where the policies explored in this study have not been implemented.

Although the use of PrEP is rising, the shape of this curve (Fig. 3) suggests that uptake may still not have reached the exponential phase of increase predicted by classical diffusion theory (Dearing and Kee 2012). In other industrialized world settings, rapid PrEP rollout has had dramatic results; in New South Wales, for example, delivering PrEP to 3700 gbMSM at risk over just 8 months led to a 25.1% reduction in new HIV diagnoses over the subsequent 12 months, and a 51.8% reduction in the gay suburbs of Sydney (Grulich et al. 2018). Given this potential benefit, it is imperative to implement policies that facilitate PrEP scale-up.

We calculated PNRs to analyze how well the need for PrEP is being met in Ontario, and, encouragingly, found that PNRs were generally most favourable (i.e., highest) among populations with the highest number of HIV diagnoses (i.e., 30–39-year-old males in Ontario’s largest city of Toronto). The overall PNR for Ontario (3.4) was higher than that observed in two US-based analyses for 2017 (1.8–2.5), despite earlier rollout of PrEP in the United States. In all cases, the largest gap in PrEP implementation was among females, who made up 21.4% of diagnoses in Ontario but only 2.5% of PrEP use. Regions neighbouring Toronto had some of the lowest PNRs, which could be due to individuals in these regions traveling to Toronto to fill prescriptions. These findings suggest the need for targeted awareness campaigns and increased PrEP providers for women and in areas outside of Toronto, along with a better understanding of the characteristics of these at-risk populations.

Regulatory approval was a crucial policy intervention for increasing PrEP use in Ontario. Notably, Health Canada approval of TDF/FTC for PrEP occurred 4 years after FDA approval in the USA, and over 5 years after publication of the iPrEx trial in which PrEP was first proven to be efficacious. This delay occurred because regulatory approvals for pharmaceutical products in Canada are contingent on the submission of an application by the manufacturer, which did not occur until 2015. Additional avenues allowing governments and other health authorities to initiate drug approvals are needed, and could improve early access to drugs that could have potentially significant public health impacts. This is particularly relevant as new antiretroviral products become available for PrEP, including injectable cabotegravir (Landovitz et al. 2019).

We also found that the addition of PrEP to Ontario’s public drug program formulary was associated with increased use, and that the percentage of users with public coverage of PrEP has been rising. Notably, listing PrEP on the public formulary increased use overall, but not among young people. This discrepancy is likely because access is contingent on preconditions being met, such as being on social assistance or payment of an annual fee. Perhaps as a result of these barriers, only 5.4% of Ontarians aged < 25 were enrolled in ODB, compared with 11.2% of people aged 25–64, and all people aged ≥ 65 (Dinh and Sutherland 2017).

In contrast, when Ontario introduced universal public drug access specifically targeted to individuals aged < 25 years, PrEP use increased. In addition, the PNR for individuals 19–24 years of age increased significantly from the end of 2017 (PNR = 1.0) to mid-2018 (PNR = 3.1), highlighting the effectiveness of policies that reduce financial barriers. The actual or perceived inability to afford PrEP is consistently cited as a barrier to PrEP use among people at risk of HIV (Bauermeister et al. 2013; Yoong et al. 2016; Sharma et al. 2014; The Resonance Project 2016). In this regard, it is unfortunate that as of April 2019, Ontario altered the OHIP+ program by disqualifying anyone with a private drug insurance plan, regardless of how much of eligible drug costs it covers.

In addition to the policy changes we evaluated, other changes occurred during the study period that may have impacted PrEP use. Canadian guidelines on PrEP prescribing were published in a widely read, Canadian general medical journal in November 2017 (Tan et al. 2017a, b), and this occurred too close in time to our other interventions of interest to be modelled separately. The significant increase detected in September 2017 may have been influenced by these guidelines. Furthermore, while we focused our analysis on provincial and national policies, local initiatives such as dedicated PrEP clinics and community-based education campaigns may have contributed to expanding PrEP use.

This study has limitations that warrant consideration. First, while we developed an algorithm to identify PrEP users as has been done previously, we were unable to validate it due to an inability to link prescription records to medical records, such that some misclassifications may exist (Mera-Giler et al. 2015). Second, double-counting of individuals in the quarterly data may have occurred if individuals filled prescriptions for TDF/FTC at more than one pharmacy in the same quarter. Third, the data do not include all retail pharmacies in Ontario, and specifically exclude prescriptions dispensed from hospitals or paid for out-of-pocket. However, the latter category is likely negligible due to the high cost of PrEP in Ontario ($875 CAD and $220 CAD per month for brand name and generic TDF/FTC respectively). Fourth, we did not assess tenofovir alafenamide/emtricitabine in our algorithm, though its use as PrEP was likely minimal since the only clinical trial demonstrating its efficacy was not presented publicly until March 2019 (Hare et al. 2019). In addition, the population-wide trends we observed may have been impacted by other secular trends that we were unable to measure. Furthermore, missing data were imputed by IQVIA, and some variables particularly related to demographics may not have been missing at random. Finally, geographic areas were based on the location of the dispensing pharmacy, not individuals’ residence. However, regions were generally large enough that the number of people living in a different region from their pharmacy was likely small.

The PNR metric has several limitations, as noted by others (Rosenberg and Marcus 2018). First, the metric uses new HIV diagnoses as a proxy for the need for PrEP, rather than incident infections, which can only be obtained through mathematical modeling. Recent modeling estimates of HIV incidence in Ontario are not available. Second, benchmark PNR thresholds above which substantial declines in HIV incidence can be observed at the population level have not been defined. While the absolute value of a PNR cannot determine how much more implementation is needed, relative comparisons of PNRs across populations can help inform the prioritization of these efforts. Third, the PNR does not indicate the clinical “appropriateness” of PrEP use, nor is there a standard regarding what level of HIV risk is sufficient to warrant PrEP. Therefore, while a PNR may be relatively high in a specific population, some people within this population may actually have relatively low HIV risk, and may be using PrEP primarily to reduce anxiety or increase sexual pleasure. Encouragingly, studies among Toronto gbMSM suggest that most individuals who seek out PrEP are indeed at elevated HIV risk (Wilton et al. 2018).

Use of PrEP in Ontario has increased following policy changes designed to expand access, and is greatest among younger, urban males, where the majority of new HIV diagnoses occur. Our findings underscore the importance of regulatory approval and public drug coverage in increasing PrEP use, and suggest that rollout in Ontario should increasingly target women and regions neighbouring Toronto.

## Electronic supplementary material

ESM 1 (DOCX 15 kb)

ESM 2 (PDF 276 kb)
