# Supplementary material for: Trends in HIV pre-exposure prophylaxis uptake in Ontario, Canada, and impact of policy changes: a population-based analysis of projected pharmacy data (2015–2018)
Source: Can J Public Health. 2020 Jun 11;112(1):89–96. doi: 10.17269/s41997-020-00332-3 (PMC7851246; doi:10.17269/s41997-020-00332-3)
Supplement: Supplementary file 1 — (DOCX 15 kb) [file 41997_2020_332_MOESM1_ESM.docx]

**Supplemental Table S2. ARIMA Model Results**

|  | **Overall** | | **Males** | | **Females** | | **Ages 24 and younger** | |
| --- | --- | --- | --- | --- | --- | --- | --- | --- |
| **ARIMA Model** | **(3,1,0) no intercept** | | **(3,1,0) no intercept** | | **(6,1,0) no intercept** | | **(3,1,0) no intercept** | |
| **R square** | **0.992** | | **0.992** | | **0.94** | | **0.985** | |
| **Interventions:** | **p-value** | **estimate** | **p-value** | **estimate** | **p-value** | **estimate** | **p-value** | **estimate** |
| February 2016 (ramp function) | 0.0001 | 31.74 | 0.0001 | 31.06 | 0.0004 | 0.51 | <.0001 | 1.94 |
| September 2017 (ramp function) | 0.0012 | 65.75 | 0.0011 | 65.27 | 0.0078 | 4.76 | 0.3197 | 1.75 |
| January 2018 (ramp function) | 0.3095 | -21.84 | 0.2458 | -24.41 | 0.2344 | 0.92 | 0.0002 | 7.79 |
| January 2018 (step function) | 0.6559 | 18.84 | 0.8145 | 9.79 | 0.0007 | 9.17 | <.0001 | 58.04 |
