# Supplementary material for: Trends in HIV pre-exposure prophylaxis uptake in Ontario, Canada, and impact of policy changes: a population-based analysis of projected pharmacy data (2015–2018)
Source: Can J Public Health. 2020 Jun 11;112(1):89–96. doi: 10.17269/s41997-020-00332-3 (PMC7851246; doi:10.17269/s41997-020-00332-3)

**SUPPLEMENTARY MATERIAL**

**Trends in HIV pre-exposure prophylaxis uptake in Ontario, Canada and impact of policy changes: A population-based analysis of projected pharmacy data (2015-2018)**

Journal name: Canadian Journal of Public Health

Darrell H. S. Tan[1234], Thomas M. Dashwood[3], James Wilton[5], Abigail Kroch[5], Tara Gomes[46], Diana Martins[4]

1. Division of Infectious Diseases, St. Michael's Hospital, Toronto, Canada
2. MAP Centre for Urban Health Solutions, St. Michael's Hospital, Toronto, Canada
3. Department of Medicine, University of Toronto. Toronto, Canada.
4. Li Ka Shing Knowledge Institute, St. Michael's Hospital, Toronto, Canada
5. Ontario HIV Treatment Network, Toronto, Canada
6. Leslie Dan Faculty of Pharmacy, University of Toronto. Toronto, Canada.

Contact information for corresponding author:

Darrell H. S. Tan, 30 Bond St., Toronto ON, M5B 1W8

Darrell.tan@gmail.com, Tel (416) 864-5568, Fax (416) 864-5310

**Table S1.** ARIMA model for time series analysis of PrEP use in Ontario, 2015 Q3 to 2018 Q2

| | **Overall** | **Males** | **Females** | **Age 24 and younger** |
|---|---|---|---|---|
| **ARIMA Model** | **(3,1,0) no intercept** | **(3,1,0) no intercept** | **(6,1,0) no intercept** | **(3,1,0) no intercept** |
| R square | 0.992 | 0.992 | 0.94 | 0.985 |
| February 2016 p-value (ramp) | 0.0001 | 0.0001 | 0.0004 | <.0001 |
| September 2017 p-value (ramp) | 0.0012 | 0.0011 | 0.0078 | 0.3197 |
| January 2018 p-value (ramp) | 0.3095 | 0.2458 | 0.2344 | 0.0002 |
| January 2018 p-value (step) | 0.6559 | 0.8145 | 0.0007 | <.0001 |

**Table S2**. Regions used and corresponding Ontario Public Health Units

| **Regions used** | **Corresponding Public Health Units** |
|---|---|
| Central East | Haliburton, Kawartha, Pine Ridge<br>Peterborough<br>Simcoe |
| Durham | Durham |
| Peel | Peel |
| York | York |
| Central South | Brant<br>Haldimand-Norfolk<br>Hamilton-Wentworth<br>Niagara |
| Central West | Waterloo<br>Wellington-Dufferin |
| Halton | Halton |
| Eastern | Eastern Ontario<br>Hastings-Prince Edward<br>Kinston-Frontenac<br>Leeds-Grenville-Lanark<br>Renfrew |
| North West | Northwestern<br>Thunderbay |
| North East | Algoma<br>North Bay<br>Porcupine<br>Sudbury<br>Timiskaming |
| Ottawa | Ottawa |

| | |
|---|---|
| Erie-St.Clair | Chatham-Kent<br>Sarnia-Lambton<br>Windsor-Essex |
| South West | Elgin-St. Thomas<br>Grey Bruce<br>Huron<br>Middlesex-London<br>Oxford<br>Perth |
| Toronto | Toronto |

**Figure S1**. PrEP use over time, stratified by (A) Payment coverage and (B) Prescriber specialty

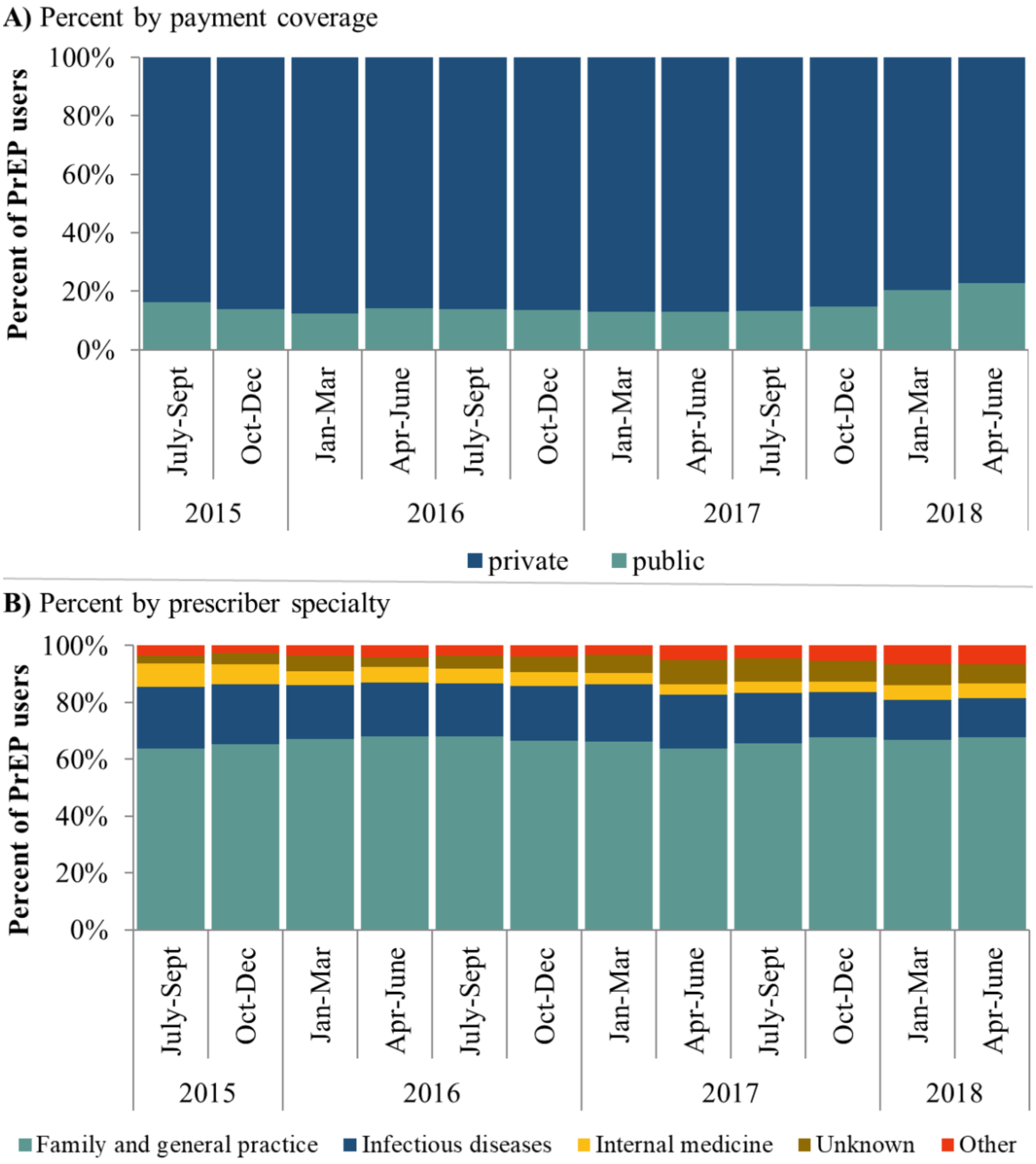

Supplement: Supplementary file 2 — (PDF 276 kb) [file 41997_2020_332_MOESM2_ESM.pdf]
